# Transcriptional responses to hyperplastic MRL signalling in *Drosophila*

**DOI:** 10.1098/rsob.160306

**Published:** 2017-02-01

**Authors:** Vincent Jonchère, Nada Alqadri, John Herbert, Lauren Dodgson, David Mason, Giovanni Messina, Francesco Falciani, Daimark Bennett

**Affiliations:** 1 Department of Biochemistry, University of Liverpool, Crown Street, Liverpool L69 7ZB, UK; 2 Centre for Cell Imaging, University of Liverpool, Crown Street, Liverpool L69 7ZB, UK; 3 Centre for Computational Biology and Modelling (CCBM), Institute of Integrative Biology, University of Liverpool, Crown Street, Liverpool L69 7ZB, UK

**Keywords:** *Drosophila*, hyperplastic growth, wing development, serum response factor, MRL proteins

## Abstract

Recent work has implicated the actin cytoskeleton in tissue size control and tumourigenesis, but how changes in actin dynamics contribute to hyperplastic growth is still unclear. Overexpression of Pico, the only *Drosophila* Mig-10/RIAM/Lamellipodin adapter protein family member, has been linked to tissue overgrowth via its effect on the myocardin-related transcription factor (Mrtf), an F-actin sensor capable of activating serum response factor (SRF). Transcriptional changes induced by acute Mrtf/SRF signalling have been largely linked to actin biosynthesis and cytoskeletal regulation. However, by RNA profiling, we find that the common response to chronic *mrtf* and *pico* overexpression in wing discs was upregulation of ribosome protein and mitochondrial genes, which are conserved targets for Mrtf/SRF and are known growth drivers. Consistent with their ability to induce a common transcriptional response and activate SRF signalling *in vitro*, we found that both *pico* and *mrtf* stimulate expression of an SRF-responsive reporter gene in wing discs. In a functional genetic screen, we also identified *deterin*, which encodes *Drosophila* Survivin, as a putative Mrtf/SRF target that is necessary for *pico*-mediated tissue overgrowth by suppressing proliferation-associated cell death. Taken together, our findings raise the possibility that distinct targets of Mrtf/SRF may be transcriptionally induced depending on the duration of upstream signalling.

## 1. Introduction

### 1.1. Actin cytoskeleton and transcriptional regulation

The actin cytoskeleton has emerged over the last few years as an important regulator of gene expression, with actin being involved in the direct regulation of transcription complexes but also in the transduction of signals to downstream transcriptional responses via the serum response factor (SRF) and Hippo signalling pathways [1]. In actin-induced SRF signalling, the SRF cofactor myocardin-related transcription factor (Mrtf) responds to levels of monomeric (G)-actin, which inhibits nuclear import and enhances nuclear export of Mrtf and represses transcriptional activation of SRF target genes [2]. Correspondingly, actin regulators that drive F-actin formation, including Ena/VASP [3], release the inhibition of Mrtf by G-actin and activate SRF. Increased levels of F-actin (e.g. mediated by anti-Capping protein or by the nucleation factor Diaphanous) also stimulate Hippo target gene expression [4,5] via the transcriptional coactivator Yorkie/YAP/TAZ, which is normally inhibited by Hippo signalling. Correspondingly, disruption of F-actin has been shown to exclude YAP from the nucleus and suppress its transcriptional activation [6–8]. Despite similarities between the regulation of Yorkie/YAP/TAZ and Mrtf-SRF by the actin cytoskeleton, expression of a mutant actin that cannot polymerize inhibits Mrtf signalling [9,10] but has no effect on YAP/TAZ [7], suggesting that the Hippo pathway is not affected by G-actin, but rather might respond to changes in a particular F-actin structure [6,7].

Results obtained in a range of species support the idea that MRL (Mig-10; RIAM; Lamellipodin) proteins act as molecular scaffolds for Ras-like GTPases and actin regulators, including Ena/VASP and the Scar/WAVE complex, to remodel the actin cytoskeleton, linking extracellular signalling to changes in cell adhesion and migration [11–13]. Recent evidence suggests that MRL proteins also play a role in growth control. For instance, in mammalian cells, Lpd knockdown abrogates the effect of EGF on proliferation [14], and abolishes Rac-induced cyclin D accumulation and mechanosensitive cell cycling [15]. In *Drosophila*, the MRL orthologue, encoded by *pico*, is capable of driving growth of the developing wing, by inducing a coordinated increase in cell size and number [14,16]. Genetic experiments in *Drosophila* have linked *pico*-mediated tissue overgrowth to activation of Mrtf/SRF as overexpression of *mrtf* or *blistered*, which encodes *Drosophila* SRF, induced tissue overgrowth and loss of function in *blistered*, and suppressed the effect of *pico* on wing size [14,17]. However, the Mrtf/SRF pathway has predominantly been associated with the expression of cytoskeletal- rather than growth-promoting genes in other contexts [18,19]. Consequently, it is not clear which genes might be induced to drive hyperplastic tissue growth and to what extent the transcriptional response to MRL protein overexpression is elicited by the Mrtf–SRF pathway.

Here we have used digital transcriptomics to determine the transcriptional responses to hyperplastic MRL signalling in the *Drosophila* wing imaginal disc. We found little evidence for involvement of the Hippo pathway in *pico*-induced overgrowth, based on minimal effect on the genes used as readouts of pathway activation. Through analysis of the Mrtf-induced transcriptome in wing discs, we identify a common signature representing possible targets of a Pico–Mrtf signalling pathway, with an *in vivo* reporter confirming the ability of *mrtf* and *pico* to drive SRF activation. Although there were clear differences in the transcriptional responses to *pico* and *mrtf* overexpression, notably, we did not see an enrichment of cytoskeletal genes in either condition. Instead, the common transcriptional signature, associated with *mrtf* and *pico*-mediated hyperplastic growth, includes ribosomal protein and mitochondrial genes that are known to be associated with Mrtf/SRF in mammalian cells, but whose significance to Mrtf/SRF function had not established. Functional analysis supports the involvement of a selection of these genes in growth control including the *Drosophila* Survivin orthologue, encoded by *deterin*, which is required for suppressing apoptosis in discs overexpressing *pico*.

## 2. Results

### 2.1. Genome-wide transcriptional changes observed in *Drosophila* wing discs with *pico* overexpression

Overexpression of *pico* with *MS1096-GAL4* (*MS1096>pico*) in the developing larval wing imaginal disc leads to a significant overgrowth of the adult wing [14]. To identify molecular signatures of ectopic *pico* expression, we micro-dissected wing imaginal discs from *MS1096>pico* and control (*w^1118^*) third instar larvae and determined their mRNA profiles by RNA-seq. For these experiments, four biological replicates were prepared from each strain. A comparison of the overall gene expression profiles of the *MS1096>pico* and control lines is shown in electronic supplementary material, figure S1. Hierarchical clustering of the replicates shows close agreement between the different samples of each line (electronic supplementary material, figure S1). Using Cufflinks [20], we identified a total of 1490 differentially expressed genes (10.7% of 13 895) in wing discs ectopically expressing *pico* (*p* < 0.05; electronic supplementary material, table S1), with 691 and 799 genes under- and overexpressed, respectively.

**Figure 1.**
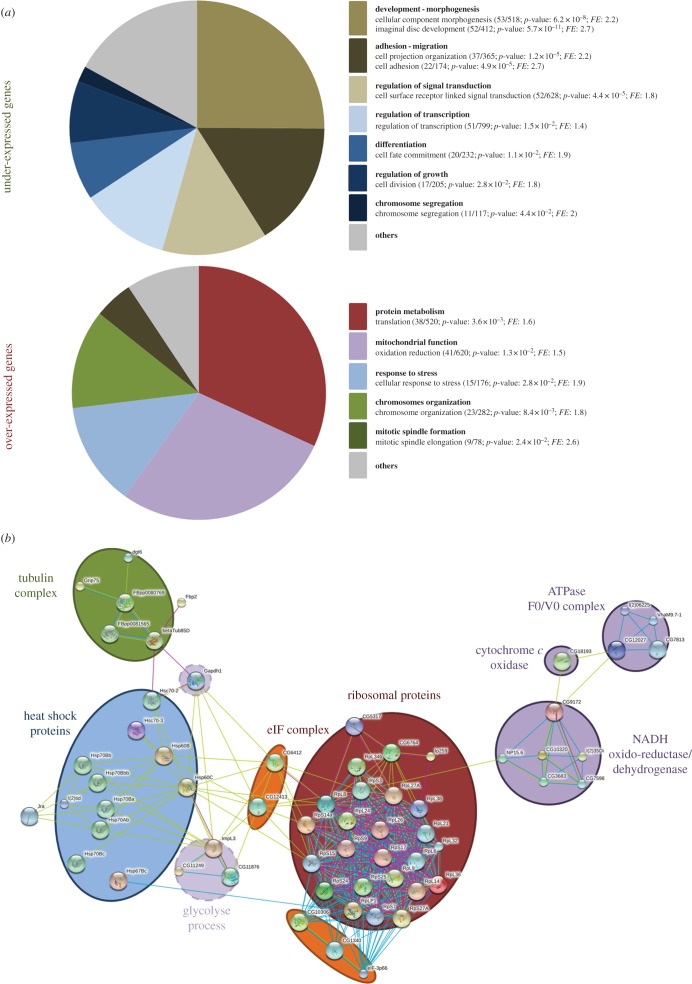
Transcriptome analysis of wing discs overexpressing pico. (*a*) Pie chart showing relative abundance of gene ontology (GO) term enrichment in genes underexpressed and overexpressed in wing imaginal discs of *MS1096>pico* third instar larvae relative to abundance of GO terms for all genes in the genome as determined by DAVID. For each category of functional group (in bold), the most prominent biological function (in italic) has been annotated with the number of genes affected in that category, the total number of genes in that category, the statistical significance (*p*-value) of the match and fold enrichment (FE). The most prominent GO categories among those upregulated in response to *pico* overexpression are related to protein biosynthesis, including initiation of translation, ribosomal function and protein maturation. There is also an enrichment in proteins localized to mitochondria. (*b*) Predicted interacting network for genes over-represented in response to *pico* overexpression, visualized using STRING. Potential associations are indicated by the links in the graph and colour coded by type: co-citation from the abstract of scientific literature (green), proteins related in curated databases (blue) and physical protein-protein for interaction databases (pink).

**Figure 2:**
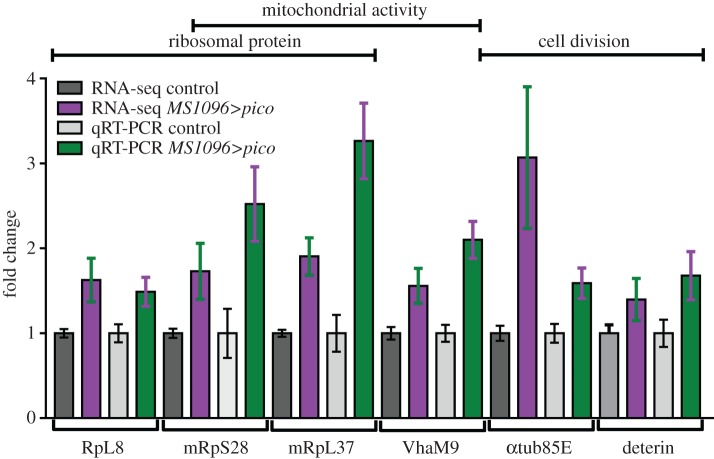
Validation of RNA-Seq by qRT-PCR. Expression levels of selected genes from *MS1096>pico* wing discs from third instar larvae, relative to control, determined by qRT-PCR and by RNA-seq. Error bars represent the s.e.m. of at least three biological replicates. The GO categories to which the genes belong are shown at the top. Individual *t*-tests without adjustment for multiple comparisons showed a significant difference (*p* < 0.05) in each case between transcript levels in *MS1096>pico* and control discs.

To identify biological processes that might be affected by ectopic *pico*, we compared the frequency of GO terms (GO) among differentially expressed genes using DAVID [21]. A large number of terms (193) were enriched among the underexpressed genes, making it hard to interpret the functional significance of these descriptors. By contrast, only 23 GO terms for biological functions were enriched among genes overexpressed in *MS1096>pico* wing discs belonging to five main categories (figure 1*a*), with translation and chromosome organization being the most significant (*p* = 3.6 × 10^−3^ and *p* = 8.4 × 10^−3^, respectively). *Drosophila*-specific searches with FlyMine also revealed enrichment of ribosome pathways (*p* = 2.7 × 10^−6^, Holm–Bonferroni). We used STRING [22] to help visualize overexpressed protein networks, which revealed 6 key network hubs genes overexpressed in response to ectopic *pico* (figure 1*b*): ribosomal proteins, eukaryotic initiation factors (eIFs), heat-shock proteins, tubulins, mitochondrial proteins and proteins involved in glycolysis. These patterns of transcriptional change are consistent with the wing overgrowth phenotype exhibited by animals with ectopic *pico*. To confirm our RNA-seq results, we selected genes representative of enriched GO categories for quantitative real-time qRT-PCR analysis. Measurements of relative mRNA expression level determined using this method were in close agreement with RNA-seq data. Indeed, even genes that that were only modestly overexpressed (approx. 1.5-fold) by RNA-seq were found to be significantly increased when assessed by qRT-PCR (*p* < 0.05; figure 2). The transcriptome dataset therefore accurately captures the expression profile of hyperplastic tissues and contains genes that promote overgrowth induced by ectopic *pico*.

### 2.2. Pico is capable of inducing SRF-responsive gene expression *in vivo*

**Figure 3.**
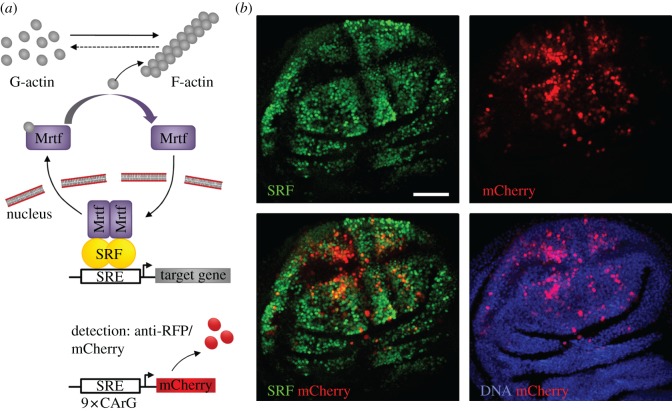
Distribution of an *in vivo* SRF-responsive reporter gene in wing discs. (*a*) Model for Mrtf/SRF activation by Pico overexpression. Increased F-actin formation leads to sequestration of G-actin, relieving inhibition of Mrtf, which translocates to the nucleus and complexes with SRF to drive transcription of genes containing SRE. SRF activation can be monitored using an SRE-mCherry reporter. (*b*) Confocal images of wing discs harbouring SRE-mCherry transgenic reporter, stained with anti-SRF antibody. The distribution of the SRE-mCherry reporter closely matches the distribution of SRF protein in presumptive intervein cells.

Actin dynamics have been reported to induce Hippo signalling, a tumour suppressor network regulating growth [5,19]. However, the absence of any induction of canonical Hippo targets in response to ectopic *pico* suggests that the *pico*-mediated growth is unlikely to be driven by engagement of the Hippo pathway (electronic supplementary material, figure S2). We previously reported that *mrtf* overexpression phenocopied the effect of ectopic *pico* in the wing and *pico*-mediated overgrowth was sensitive to the levels of *blistered*, suggesting that tissue overgrowth might require SRF-dependent gene expression [14]. To test the ability of *pico* overexpression to induce SRF signalling in the wing disc, we generated transgenic flies harbouring an *in vivo* SRF reporter, consisting of an SRF-responsive element (SRE), containing nine CArG binding motifs (CC[A/T]_6_GG), upstream of the coding sequence for mCherry (SRE-mCherry) (figure 3*a*). In wing discs from third instar larvae, expression of SRE-mCherry was often very weak, but recapitulated the expression pattern of SRF protein, which is restricted to intervein cells (figure 3*b*). Although relatively few SRF-expressing cells expressed the mCherry reporter, 93.1 ± 10.6% (mean ± s.d., *n* = 5 discs) of cells with detectible SRE-mCherry expressed SRF. Stronger expression of the reporter was detected as the wing disc matured; by pupariation, in animals with two copies of the reporter, mCherry was clearly visible in the pupal wing but not other tissues.

**Figure 4.**
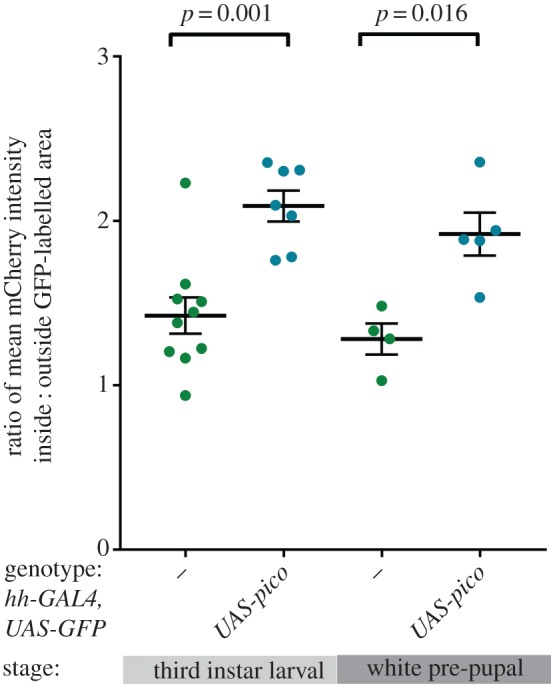
Overexpression of *pico* induces SRE-mCherry expression in larval and pupal wing discs. Scatterplot shows measurements from different wing imaginal discs of the ratio of mean mCherry intensity in cells inside : outside the GFP-labelled posterior half of each disc (from z-stacks of at least four wing discs per genotype). Mean values ±s.e. for each genotype are indicated with a line. The genotype and developmental stage are as indicated (dashes correspond to *hh-GAL4*, *UAS-GFP* alone). The results of *t*-tests comparing mCherry levels between discs with or without overexpressed *pico* at each stage are indicated.

**Figure 5.**
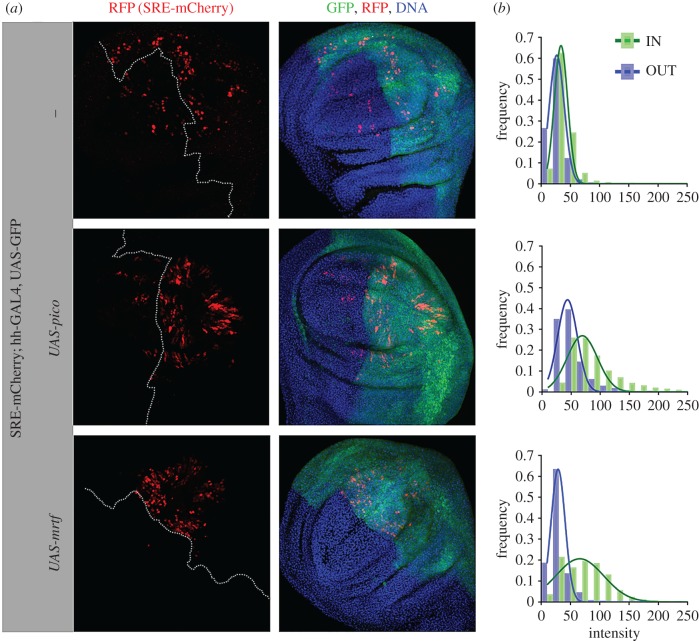
Overexpression of *mrtf* or *pico* induces SRE-mCherry expression. (*a*) Shown are representative images of an apical view of wing discs overexpressing *mrtf* or *pico* in the posterior compartment of third instar wing imaginal discs (marked with GFP) using *hh-GAL4*. GFP labels *hh-GAL4* expressing cells (in green), anti-RFP antibody staining reveals SRE-mCherry distribution (in red), TOPRO-3 staining reveals DNA (in blue). For clarity, a dotted line in the images showing SRE-mCherry alone indicates the position of the anterior/posterior boundary. (*b*) *mrtf* and *pico* induce SRE-mCherry expression *in vivo*. Shown are graphs of the distribution of SRE-mCherry signal intensities inside (IN) or outside (OUT) GFP-labelled compartments (from z-stacks of at least 10 wing discs). Levels of SRE-mCherry were noticeably elevated in the posterior half of discs expressing *mrtf* or *pico*.

To test the effect of *pico* on the expression of our reporter gene, we overexpressed *UAS-pico* in the posterior half of the wing disc, together with UAS-GFP, under the control of *hh-GAL4*, and measured the ratio of mCherry fluorescence in anterior (GFP-negative) : posterior (GFP-positive) cells (figures 4 and 5). To ensure signal intensities were in the linear range, laser power was adjusted so that the maximum-intensity signal was below the level of saturation for the detectors. Cells in control discs from third instar larvae showed a mean anterior : posterior mCherry ratio of 1 : 1.4, reflecting the fact there are more intervein cells in the posterior half of the disc. There was a 1.5-fold increase in the mean ratio of mCherry intensity (to 1 : 2.1) in wing discs overexpressing *pico* compared with the controls (*p* = 0.001). A similar induction (of 1.5-fold) in SRE-mCherry expression was seen in wing discs from white pre-pupae, indicating this effect was not stage specific (figure 4). We also confirmed this effect by pooling intensity measurements from multiple discs and comparing the intensity bias in GFP and non-GFP compartments (figure 5*b*). This revealed a 1.4-fold increase in the mean intensity of mCherry in the presence of overexpressed *pico* compared with controls (*x*^2^ < 0.001). A similar effect was observed for overexpressed *mrtf* (mean fold change 1.9, *x*^2^ < 0.001). These data indicate that both *mrtf* and *pico* are capable of inducing the SRE-mCherry reporter gene in the wing imaginal disc, consistent with their reported effects on SRF signalling in mammalian cells [14,23].

### 2.3. Upregulation of ribosome and mitochondrial genes is a common response to overexpressed *pico* and *mrtf*

To identify potential targets of Mrtf/SRF signalling that might be subject to regulation by *pico*, we analysed the mRNA expression levels of wing discs overexpressing wild-type *mrtf* (*MS1096>mrtf*) by RNA-seq. This revealed a total of 1526 differentially expressed genes (11% of 13 895) in wing discs ectopically expressing *mrtf* compared with *w^1118^* controls (*p* < 0.05, electronic supplementary material, table S1), with 820 and 706 genes under- and overexpressed, respectively. A comparison of the overall gene expression profiles of the *MS1096>mtrf* lines and hierarchical clustering of the replicates is shown in electronic supplementary material, figure S1. Interestingly, we did not observe a significant induction of Actin5C, which has been proposed to be a homeostatic target of Mrtf/SRF in ovaries [24], nor did we see enrichment of GO categories corresponding to cytoskeletal genes among the overexpressed genes in DAVID or FlyMine (electronic supplementary material, table S2).

**Figure 6.**
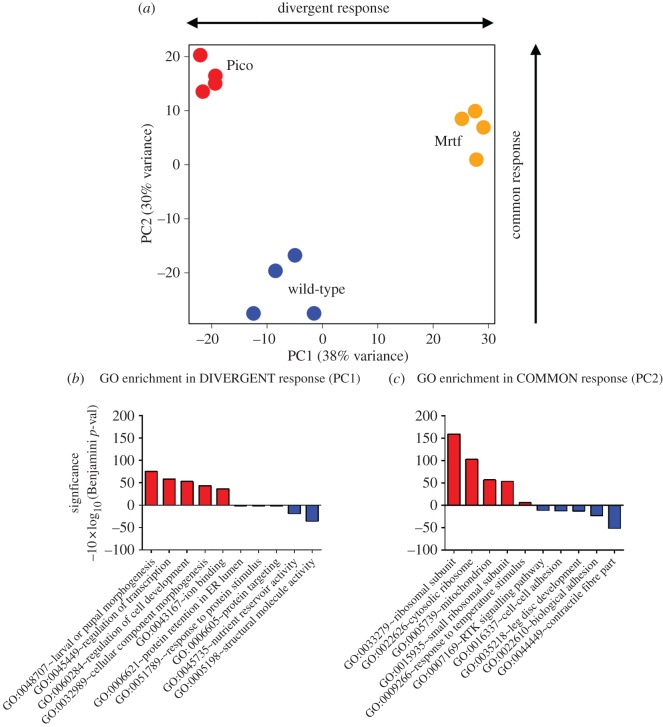
Divergent and common responses to *mrtf* and *pico* overexpression. (*a*) Principal component analysis showing divergent (PC1) and common (PC2) response to overexpressed *pico* and *mrtf*, respectively, which together explain approximately 70% of the variance in gene expression. Data points are four independent biological repeats for each condition (*MS1096>pico*, red; *MS1096>mrtf*, yellow, *MS1096-GAL4* alone, blue). (*b*,*c*) GO enrichment for the top (red) and bottom (blue) 10% of loadings from divergent and common responses. The five most significant GO categories for each grouping are shown. Both *pico* and *mrtf* overexpression stimulate ribosome protein genes belonging to the GO category ‘ribosome subunit’.

To determine the relationship between the effects of *mrtf* and *pico*, we subjected our RNA-seq datasets to principal component analysis, which is not subject to thresholding and therefore has the ability to scrutinize all the available transcriptomics data in a non-biased fashion. We took this approach because we reasoned that Mrtf-SRF-responsive genes might show modest changes in transcript levels (similar to our reporter gene), but be biologically relevant when individual genes belong to a cohort of genes with related functions that show a consistent change in expression. Principal component analysis identified a divergent (PC1) and common (PC2) response to overexpressed *pico* and *mrtf*, respectively, which together explain approximately 70% of the variance in gene expression (figure 6*a*). Clustering of the biological replicates for each genotype indicated these responses are highly reproducible.

To determine whether there was any enrichment of genes belonging to functionally related biological processes, we analysed the distribution of GO terms in the PC1 and PC2 loadings. The signatures associated with the divergent response related to broad GO terms such as morphogenesis, transcription and regulation of cell development (figure 6*b*). Literature mining of PC1 components identified a significant enrichment in circadian clock genes (*p* = 3.3 × 10^−8^), with 76 Clock target genes [25] being associated with the divergent response. Correspondingly, we found differential expression of mRNA for core clock components *timeless* and *clock*, but not *period*, whose human orthologue is a target of Mrtf [19]. This suggests that part of the divergent response might be explained by alterations in phasing of the circadian clock.

Strikingly, the overexpression of ribosome protein and mitochondrial protein genes were highly significant common responses to *pico* and *mrtf* overexpression (figure 6*c*). We wondered whether ribosome genes representing part of the common transcriptional signature (PC2) for *mrtf* and *pico* may be direct targets of Mrtf. To assess this we referred to a dataset of genes known to be bound by Mrtf in *Drosophila* adult ovaries based on ChIP-Seq [24]. We examined the overlap between the top 10% of Mrtf-binding sites ranked according to their *p*-value score [24], and the top 10% of upregulated genes in PC2. Contained in this dataset are a total of 19 ribosome protein genes representative of the enriched GO category in PC2. As ribosome protein genes are highly conserved, we were also able to ask whether these genes represent targets of Mrtf in mammalian cells. Ninety per cent of homologous genes were found to be experimentally associated with both SRF and Mrtf in fibroblasts [19].

### 2.4. Identification of differentially expressed genes contributing to *pico*-mediated overgrowth by directed screening

**Figure 7.**
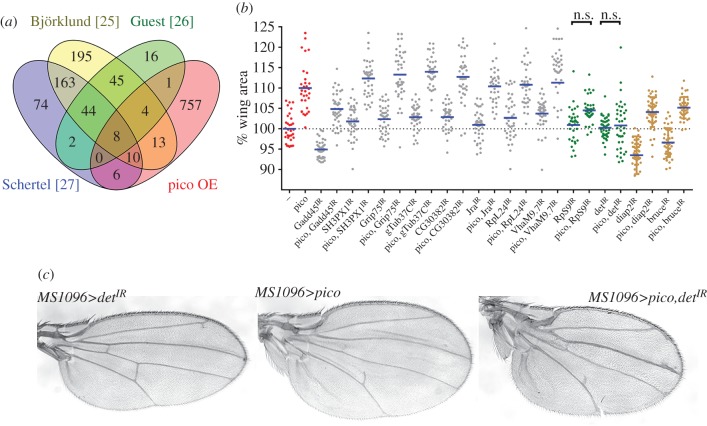
Genes induced by *pico* overexpression are required for *pico*-mediated tissue growth. (*a*) Venn diagram showing overlap between genes significantly overexpressed by *pico* (identified in this study) and genes identified for their role in the cell cycle or growth control in high-throughput functional genetic studies [26–28]. (*b*) Results of genetic screen showing effect of inverted repeat (IR) constructs for 12 genes on adult male wing size, with or without overexpressed *pico*, expressed as a percentage of control (first column shows *MS1096-GAL4* alone). All strains contained *MS1096-GAL4* to drive expression in the developing wing disc. Data are shown as scatterplot and mean values indicated with a line (*n* ≥ 30). Overexpression of *pico* induced approximately a 10% increase in wing size compared with the control (red data points). Most IR constructs had little effect although *Gadd45^IR^*, *diap2^IR^* and *bruce^IR^* had a modest effect on wing size in both the presence and absence of overexpressed *pico*. Notably, *UAS-RpS9^IR^* and *UAS-det^IR^* significantly suppressed pico-mediated overgrowth but had little effect in an otherwise wild-type background (n.s., not significant, *p* > 0.05 *t*-test). (*c*) Knockdown of *deterin* (*det*) suppressed *pico*-mediated tissue overgrowth, but not an alteration to wing shape. Flies carrying a UAS-inverted repeat for *deterin* under the control of *MS1096-GAL4* (*MS1096>det^IR^*) resembled wild-type wings (not shown).

To identify potential effectors of *pico*-mediated tissue overgrowth, we compared the overlap of our transcriptomics data with functional information from three publications [26–28] describing 651 genes involved in cell-cycle progression or growth in *Drosophila* (figure 7*a*). This identified 42 *pico*-responsive genes previously demonstrated to regulate cell-cycle progression or cell growth. Of these proteins, 45% (19/42) are ribosomal proteins, two belong to tubulin family (α, β tubulin) and one is a subunit of eIF3 protein (eIF3p66), all of which are representative of the protein hubs identified in our GO enrichment and STRING network analyses (figure 1). Next, to assess their role in *pico*-mediated overexpression, we selected 17 genes with heritable RNAi constructs in flies and measured their effect on adult wing size and morphology alone or in the background of overexpressed *pico* driven by *MS1096-GAL4* (figure 7*b*). This group of proteins included genes described above, other ribosomal proteins, tubulin proteins, mitochondrial proteins and chromosome passenger complex proteins. RNAi lines for seven genes (*VhaM9.7-c*; *CG30382*; *RpL24*; *Grip75*; *SH3PX1*; *Jra*; *γTub37C*) did not show any detectable effect on wing size; three (*Prosalpha5*; *NP15.6*; *RpL6*) caused larval lethality; four others (*RpS3*; *alphaTub85E*; *RpS27A*; *RpS17*) induced a wing dysmorphology phenotype making interpretation of effects on growth difficult. RNAi for two genes (*RpS9* and *deterin*) showed a significant reduction (*p* < 0.05) on the size of *MS1096*>*pico* wings but not *MS1096-GAL4* controls (figure 7*b,c*).

### 2.5. Deterin is required for pico-mediated tissue overgrowth

As *deterin* knockdown had the strongest effect on *pico*-mediated growth we focused our attention on this genetic interactor. *deterin* encodes *Drosophila* Survivin, a member of the inhibitor of apoptosis (IAP) gene family that has been implicated in apoptosis, chromosome segregation and cytokinesis [29,30]. First, to determine how specific the effects of *deterin* were, we examined whether knockdown of other apoptosis inhibitors, *diap2* and *bruce*, was similarly able to suppress pico-mediated growth. Notably, although both *diap2* and *bruce* knockdown were able to reduce adult wing size, this effect was independent of *pico* overexpression, suggesting that overgrowth driven by *MS1096>pico* was particularly sensitive to the levels of *deterin* and not these other apoptosis regulators (figure 7*b*).

**Figure 8.**
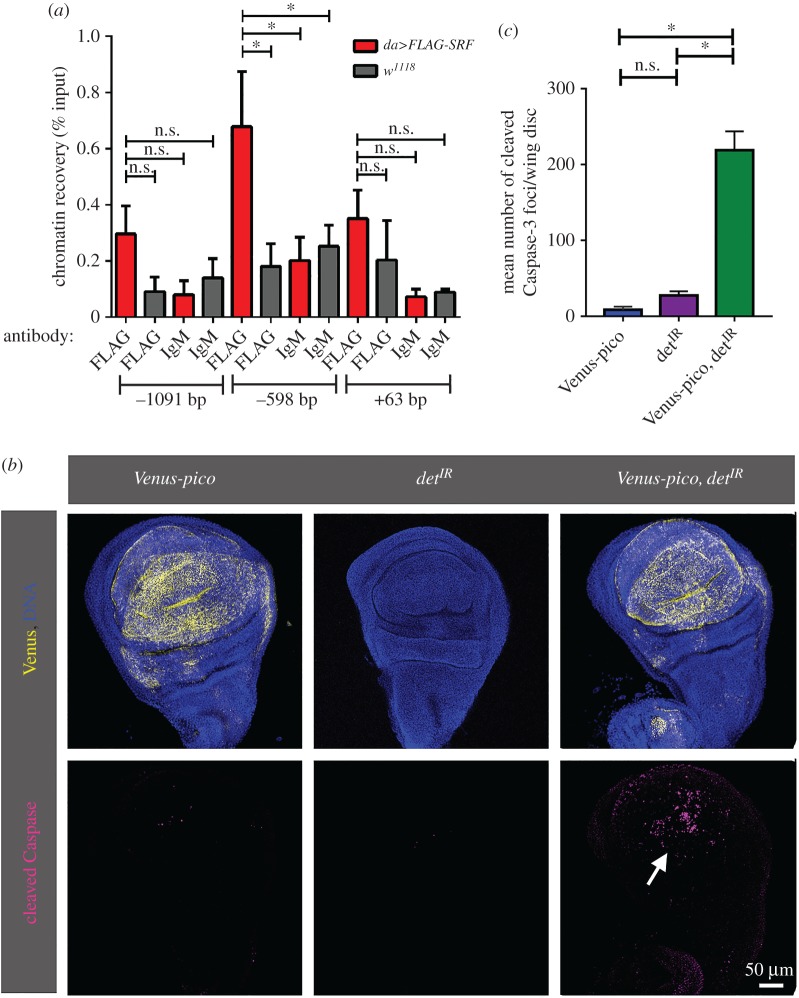
Deterin is a Mrtf/SRF target and suppresses apoptosis during *pico*-mediated tissue overgrowth. (*a*) A site 5′ of the transcription start site (TSS) of *deterin* binds FLAG-SRF. Chromatin immunoprecipitation (ChIP) analyses of three sites at the 5′ end of *deterin* containing a potential CArG box. Position of the CArG box relative to the TSS is indicated at the bottom. ChIP from third instar larval *da>Flag-SRF* and control larvae (*da-GAL4* alone) was performed using monoclonal anti-FLAG and mouse IgM antibodies. Immunoprecipitated DNA was quantified by qPCR. For each genotype, percentage input is the amount of precipitated DNA relative to input DNA. Results are mean ± s.e.m. from three independent experiments. One-way ANOVA: *p < 0.05; n.s., not significant. Distance of sites from the TSS is indicated on the *x*-axis. (*b*) *det^IR^* induces cell death in wing discs co-overexpressing *pico*. Discs overexpressing Venus-pico (in yellow) are overgrown and show little cleaved Caspase-3-staining (in red); coexpression of detIR reduced tissue size and induced cleaved Caspase-3 in the centre of the wing pouch (arrow). (*c*) Quantitation of number of Caspase-3-positive foci in wing discs expressing *Venus-pico* or *det^IR^*, alone or in combination (mean ± s.e.m. from z-stacks of at least three wing discs). *t*-test: *p < 0.05, n.s., not significant.

Our RNA-seq data indicated a modest increase in Det mRNA levels of up to 1.4-fold in wing disc extracts following *mrtf* overexpression (mean ± s.e. 1.2 ± 0.10, *n* = 4). qRT-PCR of carefully matched wing disc samples showed a mean fold increase of 1.46 (±0.12 s.e., *n* = 4), comparable with the response to overexpressed *pico* (figure 2). Human Mrtf and SRF have been shown to associate with the Survivin/BIRC5 promoter in fibroblasts, prompting us to examine whether *deterin* is a direct target of SRF in flies. We searched the presence of CarG boxes in the *deterin* promoter, and found three sites at −1091, −598 and +63 from the transcription start site (TSS). To test SRF binding experimentally we expressed FLAG-tagged SRF in larvae with *da-GAL4*, precipitated tagged SRF from extracts by ChIP and analysed chromatin recovery using qPCR (figure 8*a*). The site at −598 showed significant enrichment following ChIP with anti-Flag antibody compared with controls (IgM ChIP in *da>FLAG-SRF* and FLAG ChIP in *da-GAL4* extracts). A comparison with the Mrtf ChIP-Seq dataset [24] revealed an Mrtf-binding site corresponding to the SRF-binding site at −598, suggesting both SRF and Mrtf are capable of associating at this site.

Studies of other growth drivers, such as the miRNA bantam, have demonstrated that genes stimulating cell proliferation can simultaneously suppress apoptosis [31]. Therefore, we wondered whether *pico* only drives net proliferation when apoptosis is simultaneously prevented in a *deterin*-dependent manner. To test this, we examined the effect of overexpressing *pico* in wing imaginal discs with or without *deterin* RNAi knockdown (*deterin^IR^*) using *MS1096-GAL4*. The line of *deterin^IR^* that we used for these experiments (det^TRiP.GL00572^) is a 21 bp hairpin line with no predicted off-targets with matches greater than or equal to 15 bp [32]. Cells undergoing apoptosis were identified by activated Caspase 3 staining (figure 8*b*). As we had previously observed, stimulation of growth by *pico* was not associated with an increase in apoptosis. Similarly, *deterin^IR^* had little effect on its own, but in discs coexpressing *pico* we observed a dramatic increase in the number of cells undergoing cell death (figure 8*b*,*c*).

## 3. Discussion

MRL proteins are key molecules that modulate the actin cytoskeleton in response to guidance cues to effect changes in cell morphology and migration. Additionally to this role, several lines of evidence suggest that MRL proteins also play an important role in cell growth in normal and pathological conditions [15,33]. This role in growth was highlighted in *Drosophila* where *pico* overexpression resulted in a coordinated increase in cell proliferation and growth [14]. Here we have analysed global RNA expression to help delineate pathways linking the regulation of actin dynamics to tissue overgrowth.

Genetic data previously implicated Mrtf/SRF in the ability of *pico* overexpression to drive wing overgrowth. Here, using a genetic reporter, we found that *pico* is capable of activating SRF-responsive gene expression in the *Drosophila* wing imaginal disc. However, although SRF targets in mammalian cells include growth-promoting genes [34], the response to Mrtf/SRF activation, at least over short time periods, predominantly involves the induction of genes involved in actin filament dynamics, cell adhesion and extracellular matrix (ECM) synthesis [19]. Given this, how does excessive Mrtf/SRF signalling induce hyperplastic overgrowth? Our transcriptome analysis sheds light on this question. Cytoskeletal genes were not identified in our ontology enrichment analysis as being induced either by *mrtf* or *pico* overexpression in the *Drosophila* wing. Instead, the genes belonging to the common *pico*/*mrtf* response are ontologically related to, and in some cases are, direct orthologues of genes associated with mammalian Mrtf/SRF, including mitochondrial and ribosomal protein genes. These genes are potent growth promoters in flies and humans, and our functional analysis provided evidence that at least one of these genes (Rps9) has a role in *pico*-mediated growth, although it is likely that multiple genes are involved. One such gene is *deterin*, which we propose enables *pico* to overcome proliferation-induced apoptosis and facilitate net tissue overgrowth.

Why has a growth signature not been observed to date in mammalian cells following induction of Mrtf signalling? The deterin/survivin homologue BIRC5, as well as counterparts of mitochondrial and ribosomal genes that we identified, show no difference in expression level in mammalian cells upon acute (30 min) serum-induced Rho-actin signalling [19]. However, promoters of all these genes are associated with SRF and Mrtf. One possibility is that the differences in transcriptional response may reflect the difference between short- versus long-term exposure to Mrtf/SRF induction. The fact that their *Drosophila* counterparts could be induced in wing discs with persistent overexpression of *pico* or *mrtf* might therefore reflect a difference between acute and chronic changes in actin dynamics. Although we did not identify an induction of other transcriptional regulators acting upstream of ribosome biogenesis genes, such as *Dref* [35], in response to *pico* overexpression, we cannot rule out the possibility that some of the transcriptional responses are indirect. This issue will require careful dissection of the promoter regions of candidate targets to monitor the chromatin environment, SRF/Mrtf occupancy and identify *cis* acting sequences that might confer temporal control of their expression under conditions of altered actin dynamics. It will be interesting to determine whether ‘constitutively expressed’ genes, which were initially refractory to Mrtf signalling in mammalian cells, become responsive after chronic induction and whether this similarly promotes a proliferative phenotype. Models of increased ECM rigidity might be the starting point for this analysis as ECM rigidity has been linked to chronic changes in actin dynamics and nuclear MRTF accumulation [36], as well as Rac and Lpd-dependent proliferation [15]. Furthermore, it will be interesting to examine whether changes in responsiveness to altered actin dynamics over the long term are associated with alterations to chromatin structure and local activity states.

Despite significant overlap in transcriptional responses to *pico* and *mrtf* overexpression, there was also significant divergence in the response, as identified by our principal component analysis. There could be several reasons for this. For instance, although *mrtf* and *pico* overexpression may increase nuclear accumulation of Mrtf by a G-actin titration mechanism, Mrtf dynamics may not be equivalent under the two conditions. Furthermore, differences in actin levels and/or actin dynamics may have additional effects. For instance, nuclear actin, which is constantly exchanging with a cytoplasmic pool [37], associates with the basal transcription machinery and chromatin modifying complexes to regulate chromatin remodelling, epigenetic programming and gene expression (reviewed [1]). Consequently, although both *mrtf* and *pico* are capable of activating SRF-mediated transcription as measured by our *in vivo* reporter, there are likely to be differences in the chromatin environment, and consequently Mrtf/SRF occupancy, at different native target sites that may influence target response.

What is the involvement of the Hippo pathway in *pico*-mediated overgrowth? The transcriptome obtained from wing discs overexpressing *pico* yields little evidence of Yorkie target gene activation, suggesting that F-actin levels, subcellular location and/or structures induced by *pico* do not modify Hippo pathway activity. Lack of interaction with Hippo signalling is supported by studies in S2 cells, indicating that knockdown of *pico* has little effect on expression of Yorkie-dependent gene expression [5]. These data are also consistent with other studies that have suggested that YAP-TAZ and MRTF-SRF signalling are independent of one another [7,38]. Nevertheless, it will be interesting to examine whether regulators of F-actin that activate Hippo also activate SRF and whether Mrtf/SRF-dependent gene expression contributes to Hippo-mediated overgrowth, as Mrtf activation would be anticipated under conditions of elevated F-actin. Our *in vivo* reporter will be of use in helping to dissect these questions.

In summary, our work provides additional insight into the molecular mechanisms by which actin remodelling acts as a growth-promoting feature. As the experimental conditions we have examined focus on the effects of overexpression, our findings are likely to have most relevance to abnormal states associated with excessive MRL activity or Mrtf/SRF signalling. Indeed, the transcriptome analysis we report here identifies features of human cancer found in hyperplastic *Drosophila* cells. The association between excessive ribosome biogenesis, translation capacity and proliferation of cancer cells, in particular, has been well documented [39–41].

## 4. Material and methods

### 4.1. Fly husbandry and genetics

Flies were reared at 25°C under standard conditions. For overexpression of Flag-tagged SRF, full-length SRF cDNA from 1–3 h *Drosophila* embryos was subcloned into pPFMW (Drosophila Genome Resource Center) to introduce an N-terminal Myc-Flag tag (pUAS-SRF-Flag); transgenic flies were generated by *p*-element-mediated insertion into a *w^1118^* strain. To make the SRE-mCherry reporter strain, a synthetic DNA sequence containing nine consensus CArG boxes (electronic supplementary material, figure S1 and text S1), was inserted into the *Not*I/*Kpn*I sites in pRedRabbit [42]; transgenic flies were generated by φC31 Integrase-mediated transgenesis, with insertion into the *attP18* landing site.

### 4.2. Genotypes

#### 4.2.1. RNA-Seq

*w, MS1096-GAL4/w^1118^* (*MS1096*)

*w, MS1096-GAL4/w^1118^;; UAS-HM-pico/+* (*MS1096>pico*)

*w, MS1096-GAL4/w^1118^; UAS-mrtf/+* (*MS1096>mrtf*)

#### 4.2.2. SRE-mCherry reporter experiments

*w, SRE-mCherry; hh-GAL4, UAS-GFP/+* (*SRE-mCherry, hh>GFP*)

*w, SRE-mCherry; hh-GAL4, UAS-GFP/UAS-mrtf* (*SRE-mCherry, hh>GFP, mrtf*)

*w, SRE-mCherry; hh-GAL4, UAS-GFP/+; UAS-HM-pico/+* (*SRE-mCherry, hh>GFP, pico*)

#### 4.2.3. ChIP

*da-GAL4/+* (*da*)

*da-GAL4/+; UAS-FLAG-SRF* (*da>SRF*)

#### 4.2.4. Analysis of adult wing size

*MS1096>UAS-pico* with *UAS-gene^IR^ (on 2 or 3)/Tft; /MKRS*

*MS1096>UAS-pico* with *Tft and MKRS*

Details of inverted repeat lines used for RNAi are provided in electronic supplementary material, text S1.

#### 4.2.5. Caspase staining

w, MS1096-GAL4/w^1118^;; UAS-Venus-pico/+

w, MS1096-GAL4/w^1118^;; UAS-Venus-pico/UAS-det^IR^

w, MS1096-GAL4/w^1118^;; +/UAS-det^IR^

### 4.3. RNA-seq and bioinformatics

Third instar larval imaginal tissues were dissected in cold phosphate buffered saline buffer, put in RNAlater (Invitrogen), quickly frozen in liquid nitrogen and stored at −80°C until isolation of RNA. Four pools of imaginal discs were made for each condition tested (*MS1096-GAL4* alone, *MS1096>pico* and *MS1096>mrtf*) corresponding to at least 300 imaginal discs/pool. RNA extractions were performed using the Ambion RNAqueous-Micro Kit (Invitrogen). RNA concentrations were measured at 260 nm with NanoDrop1000 spectrophotometer (Thermofisher) and RNA integrity was assessed with Agilent 2100 Bioanalyser. mRNA was enriched from total RNA samples, using the Dynabeads mRNA purification kit from Total RNA Preps (Invitrogen). The libraries were prepared according to the ScriptSeq v.2 RNA-Seq Library Preparation Kit protocol (Epicentre). The indexed and multiplexed mRNA libraries were sequenced on an Illumina HiSeq 2000, using paired-end chemistry with 2 × 100 bp read lengths (Illumina). More than 40 million reads were generated for each sample. Reads were filtered for quality and mapped onto the *Drosophila melanogaster* reference genome version dm5.39 [43] using TopHat 2.0 [43]. The number of reads mapping to each gene were calculated using HTSeq-count [45], and the count data were further analysed using EdgeR [46]. The data were normalized to account for differences in library size, and a generalized linear model was applied, using *MS1906-GAL4* alone as the reference and contrasting this with *MS1096>pico* and *MS1096>mrtf*. *p*-values were obtained using *t*-tests, and adjusted using the Benjamini & Hochberg [47] multiple testing procedure to control the false discovery rate (FDR). An FDR cut-off of 0.05 was applied to identify differentially expressed genes. RNA-Seq data have been deposited in the NCBI Sequence Read Archive, accession no. SRP068408.

### 4.4. Gene ontology analysis

To analyse the enrichment of the genes belonging to specific biological processes, significantly down- or upregulated genes were analysed by Database for Annotation, Visualization and Integrated Discovery (DAVID) [21] against *D. melanogaster* database (*p*-value, 0.05; min. genes, 5) or using FlyMine (www.flymine.org) [48]. The protein–protein interactions were obtained using STRING [22]. Active prediction methods were experiments, databases and textmining and a medium confidence score (STRING global scores: 0.4) was applied to identified the predicted interactome, which was based on experimental evidence, database association and co-citations.

### 4.5. Principal component analysis

Principal component analysis was carried out using the open source statistical package R and the prcomp function. The loadings of the first 2 components were extracted, and the top 10% of the most positive and most negative loading genes were subject to DAVID gene-annotation enrichment and functional annotation cluster analysis [21]. Each annotation cluster was summarized into a single term, taking the most significant term from each cluster, using an FDR cut-off of 10%. These terms were displayed in a bar plot using values of −10 × log_10_ of the Benjamini & Hochburg adjusted *p*-values. Literature mining was performed using FlyMine [48].

### 4.6. qRT-PCR

qRT-PCR procedures were described previously [16]. Briefly, 1 µg of total RNA samples were subjected to reverse-transcription using high capacity RNA-to-cDNA kit (Applied Biosystems/Invitrogen). Primer design was performed using Primer3 online software [49]. cDNA were amplified in real time using the qPCR Master mix plus for power SYBR Green I assay (Invitrogen) and analysed with the StepOnePlus real-time PCR system (Applied Biosystems). The level of gene expression in extracts from *MS1096>UAS-pico*, and *MS1096>mrtf* was compared with controls (*MS1096-GAL4* alone) and expressed as a ratio. Primers used for qPCR are given in electronic supplementary material, text S1.

### 4.7. CArG predictions

The promoting sequences of each selected gene were retrieved (positions −4000 to 100 with respect to the transcriptional start site) with removing sequences shared with neighbouring genes. Using the matrix of SRF-binding sites experimentally validated in mammals [34] and our promoter sequences, a positional weight matrix were applied using the matrix-scan software [50]. The parameters used for the analysis were those given by default by the software. Binding sites with a *p* ≤×10^−4^ and a weight up to 5 were considered significant.

### 4.8. Chromatin immunoprecipitation-qPCR

ChIP experiments were performed from wandering L3 larvae, as described previously [51]. For the immunoprecipitations, 25 µg of chromatin was incubated overnight with antibody and another 4 h the next day with protein G-coated magnetic beads (Diagenode or Millipore). The antibodies used in the IP were: mouse anti-FLAG (F3165, Sigma) and mouse IgM. The DNA was recovery with Ipure Kit (Diagenode). A minimum of three biological replicates was done for each genotype. For the qPCR analysis, reactions were done in duplicates and the quantity of DNA bound by specific antibodies was calculated by percentage input. Primers used for qPCR are given in electronic supplementary material, text S1.

### 4.9. Immunofluorescence

Tissues dissected from third instar larvae were fixed and stained following [20] with primary antibodies: rabbit anti-cleaved caspase-3 (Cell Signalling Technology, 1 : 100), rat anti-RFP (Chromotek, 1 : 1000). Secondary antibody conjugated to Alexa-Fluor 555 (1 : 500) for 2 h at room temperature in the dark. TO-PRO-3 Iodide (Invitrogen, 1 : 1000) or Hoescht (1 : 1000) was used to visualize DNA. Tissues were mounted in Vectashield mounting medium (Vector Laboratories) and imaged on a Zeiss LSM780 or LSM880 microscope equipped with 405 nm, 488 nm, 568 nm and 633 nm lasers using a 20× objective or Plan Apochromat 40×/1.3NA oil immersion objective. Images were imported into Omero [52] and adjusted for brightness and contrast uniformly across entire fields where appropriate. Figures were constructed in Adobe Photoshop.

### 4.10. Image analysis

For measurements of SRE-mCherry, image analysis was conducted using Bitplane Imaris v. 8.2.0 (Oxford Instruments). The GFP channel was segmented into a single three-dimensional volume (5 µm surface grain size) by absolute intensity using an automatically selected intensity threshold. Small unattached objects were removed using a volume filter. This segmented volume was used to mask the red channel into two new channels by zeroing all voxels inside or outside the volume. These new channels were used to segment the data into spots (estimated diameter of 5 µm, using background subtraction). Spots were subjected to an automatically thresholded intensity filter, which was visually inspected and adjusted if necessary. The mean intensity of each spot was recorded for each sample. The GFP-positive fraction was calculated as the number of GFP-positive spots divided by the total number of spots. Intensity bias was calculated as the mean intensity of GFP-positive spots divided by the mean intensity of GFP-negative spots. Using the data above, the individual mean spot intensities were scaled to 8-bit values and histograms were produced using MATLAB 2015a (Mathworks). Data were binned into 13 bins spread evenly across the 8-bit range for all conditions. The histograms were then normalized by dividing all frequency values by the maximum frequency value within that dataset. Data were plotted in GraphPad, along with nonlinear regression curve assuming a Gaussian distribution.

For quantitation of cleaved Caspase 3 staining, data were segmented into spots as described above using Imaris. Spots outside the wing pouch (as observed in the transmitted channel) were removed manually, and the remaining spots were counted and their mean intensity measured.

## Supplementary Material

Fig.S1 Comparison of global RNA-Seq gene expression profiles.

## Supplementary Material

Fig.S2 Levels of Yorkie target genes in wing discs overexpressing pico.

## Supplementary Material

Table S1. Comparison of gene level expression in wings imaginal disks of pico and mrtf to control by RNA-sequencing.

## Supplementary Material

Table S2. Biological Process Ontology (BP-GO) term enrichment in genes showing their expression affected by pico and mrtf.

## Supplementary Material

Text S1. Details of RNAi lines, matrix of CarG boxes, sequence of SRE element used in SRE-mCherry reporter and primer sequences used in this paper.
