# Supplementary figures and images for: Transcriptional responses to hyperplastic MRL signalling in Drosophila
Source: Open Biol. 2017 Feb 1;7(2):160306. doi: 10.1098/rsob.160306 (PMC5356444; doi:10.1098/rsob.160306)

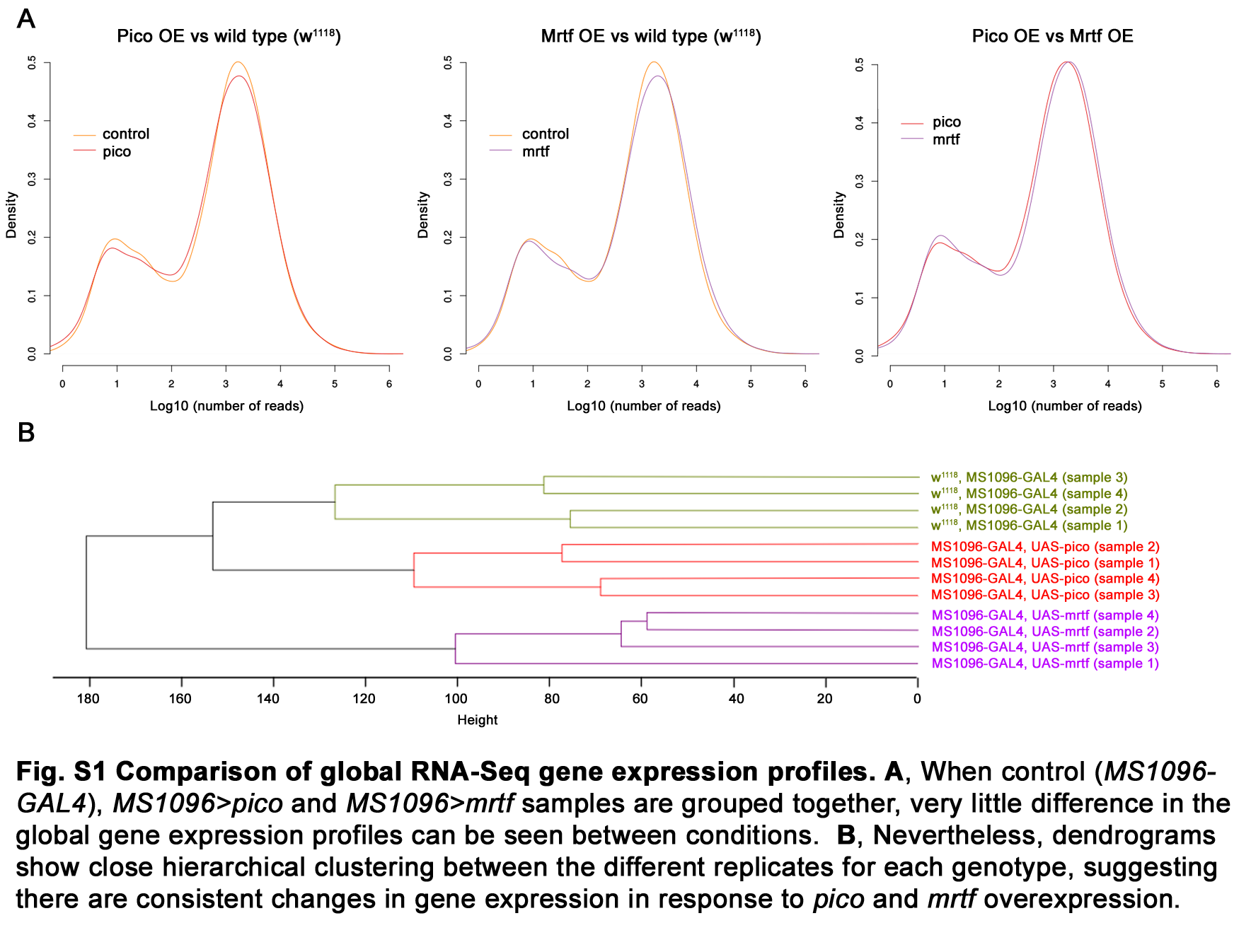

Supplement: Fig.S1 Comparison of global RNA-Seq gene expression profiles. [file rsob160306supp1.tif]

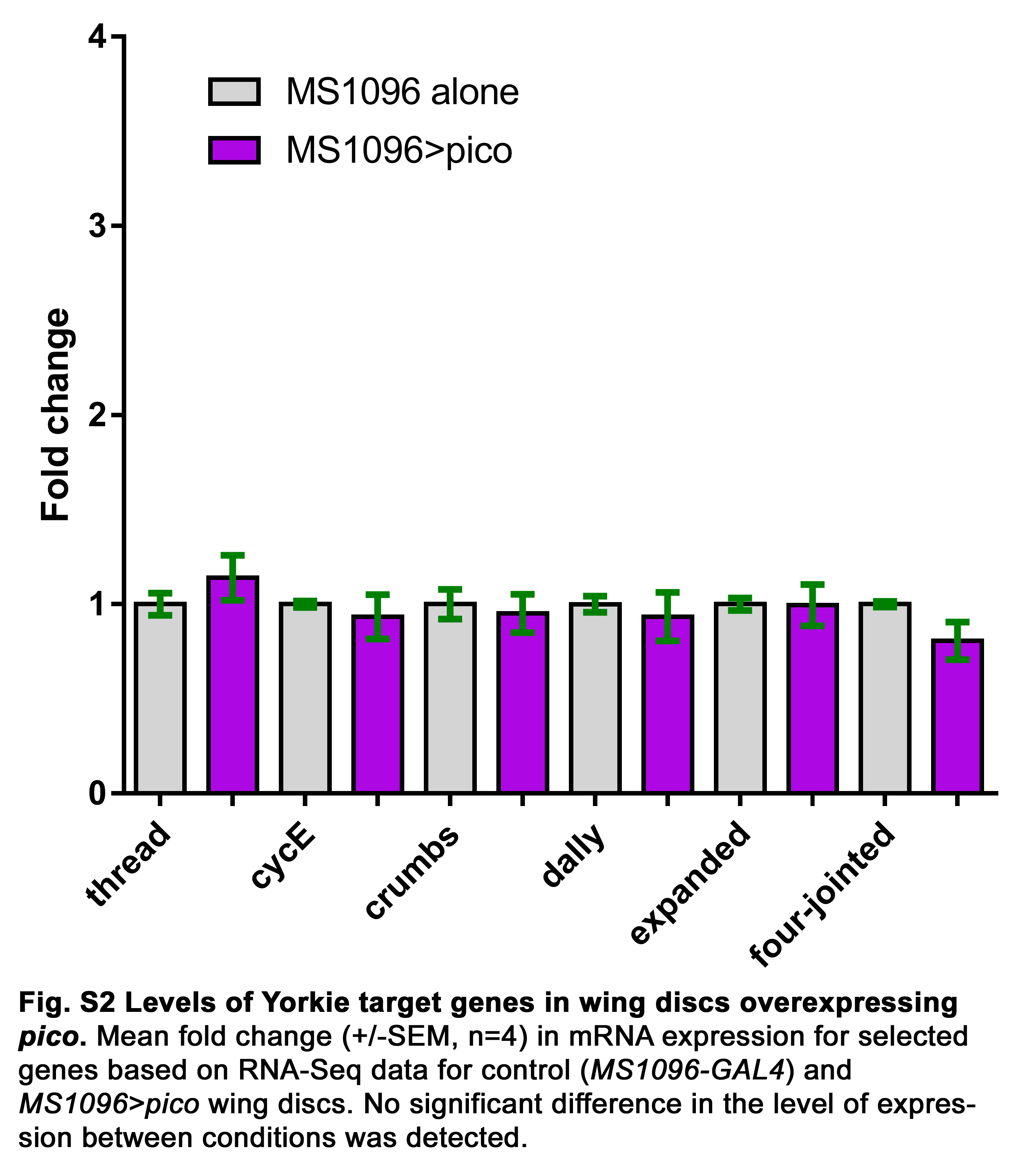

Supplement: Fig.S2 Levels of Yorkie target genes in wing discs overexpressing pico. [file rsob160306supp2.tif]
