# Supplementary material for: Transcriptional responses to hyperplastic MRL signalling in Drosophila
Source: Open Biol. 2017 Feb 1;7(2):160306. doi: 10.1098/rsob.160306 (PMC5356444; doi:10.1098/rsob.160306)
Supplement: Text S1. Details of RNAi lines, matrix of CarG boxes, sequence of SRE element used in SRE-mCherry reporter and primer sequences used in this paper. [file rsob160306supp5.docx]

**List of primers used for qRT-PCR**

| **Gene Name** | **Flybase-ID** | **Forward primer** | **Reverse primer** |
| --- | --- | --- | --- |
| RpL8 | FBgn0261602 | GTACAAGGTGAAGCGCAACAG | GCTGCAGCCAGAGCTTACTT |
| mRpS28 | FBgn0034361 | GGAAGACAACCAGACCTTCG | GACGAACCCAGAAACTTGGTG |
| mRpL37 | FBgn0261380 | CCCAAACCAATTGTTGTGCAGAG | GCGGAAGACCTCGCCATTGTAA |
| VhaM9.7-c | FBgn0028664 | TCTGGCTGTGCTGCTATTTG | GTGGTTTCACCGACTGTGTGTG |
| alphaTub85E | FBgn0003886 | GACATACGCTCCCGTCATTTC | CTGGATAGAGCGCTTGGTCTTG |
| Det | FBgn0264291 | GGAAAACGACACTGCCACTTG | GACGGTTCCAAGAATTTCCAGA |
| 18S | FBgn0085813 | CGCAAGATCGTTATATTGGTTG | GCTGCCTTCCTTAGATGTGG |

**Matrix of CArG boxes identified in mammals**

|  | *Position 1* | *Position 2* | *Position 3* | *Position 4* | *Position 5* | *Position 6* | *Position 7* | *Position 8* | *Position 9* | *Position 10* |
| --- | --- | --- | --- | --- | --- | --- | --- | --- | --- | --- |
| **A** | 1 | 1 | 57 | 32 | 78 | 26 | 75 | 52 | 7 | 2 |
| **C** | 110 | 107 | 3 | 1 | 0 | 5 | 0 | 1 | 0 | 3 |
| **G** | 1 | 0 | 2 | 0 | 8 | 4 | 3 | 1 | 105 | 105 |
| **T** | 0 | 0 | 50 | 79 | 26 | 77 | 34 | 58 | 0 | 2 |

**Sequence of SRE element in SRE-mCherry reporter (CArG boxes in underline)**

GGTACCCCGCGGGGATGTCCATATTAGGACATCTGGATGTCCATATTAGGACATCTGGATGTCCATATTAGGACATCTCTCGAGGGATGTCCATATTAGGACATCTGGATGTCCATATTAGGACATCTGGATGTCCATATTAGGACATCTAGATCTGGATGTCCATATTAGGACATCTGGATGTCCATATTAGGACATCTGGATGTCCATATTAGGACATCTCCGCGGAGATCT

**List of primers used for ChIP-PCR**

| **Gene Name** | **Flybase-ID** | **Forward primer** | **Reverse primer** |
| --- | --- | --- | --- |
| Det (box1) | FBgn0264291 | GCTGGTTTCACTGCAGACAC | GTCCCTGTAATTGGCTTCCAC |
| Det (box2) |  | GTGGGTGGTGGATGGTGTAT | GAAGAAGGAAAGGCAACCAAGC |
| Det (box3) |  | ATAACCGGGCTTACAAACTATCG | CCACTGGCGATTCCATATCCT |

**Details of RNAi lines**

| **Gene Symbol** | **Bloomington number** | **Associated function / Complex** |
| --- | --- | --- |
| Control (W1118 ) | N/A | N/A |
| Prosalpha5 | 34786 | Proteasome |
| NP15.6 | 36672 | Mitochondria |
| RpL6 | 34004 | Ribosomal protein |
| RpS3 | 31625 | Ribosomal protein |
| RpS3 | 35410 | Ribosomal protein |
| alphaTub85E | 31703 | Tubulin complex |
| RpS27A | 35418 | Ribosomal protein |
| RpS17 | 42656 | Ribosomal protein |
| gammaTub37C | 32513 | Tubulin complex |
| CG30382 | 27557 | Proteasome complex |
| VhaM9.7-c | 26004 | Mitochondria |
| CG30382 | 43202 | Proteasome complex |
| RpL24 | 34569 | Ribosomal protein |
| Grip75 | 31215 | Tubulin complex |
| SH3PX1 | 27653 | Phosphatidylinositol binding |
| Jra | 31595 | C-jun/ AP1 complex |
| Gadd45 | 35023 | Ribosomal protein |
| Rheb | 33966 | GTPase Ras superfamily |
| RpS9 | 33394 | Ribosomal protein |
| Det | 36612 | Chromosome passenger complex |
|  |  |  |
|  | **VDRC number** |  |
| bruce | 107620 | Inhibitor of apoptosis |
| dIap2 | 105351 | Inhibitor of apoptosis |
